# Carbon Nanotubes and Carbon Quantum Dots for Sustainable Agriculture

**DOI:** 10.3390/plants15192969

**Published:** 2026-09-29

**Authors:** Shuoqi Wang, Linjing Deng, Lin Wang, Qinghe Zhu, Charles Obinwanne Okoye, Jianxiong Jiang, Pei Zhou, Linchuan Fang, Xunfeng Chen

**Affiliations:** 1 Key Laboratory of Zhenjiang, School of Environment and Safety Engineering, School of Emergency Management, Jiangsu University, Zhenjiang 212013, China; 2 Key Laboratory of Urban Agriculture, Ministry of Agriculture and Rural Affairs, Shanghai Yangtze River Delta Eco-Environmental Change and Management Observation and Research Station (Shanghai Urban Ecosystem Research Station), Ministry of Science and Technology, National Forestry and Grassland Administration, School of Agriculture and Biology, Shanghai Jiao Tong University, 800 Dongchuan Road, Shanghai 200240, China; 3 Key Laboratory of Original Agro-Environmental Pollution Prevention and Control, Ministry of Agriculture and Rural Affairs (MARA)/Tianjin Key Laboratory of Agro-Environment and Agro-Product Safety, Agro-Environment Protection Institution, MARA, Tianjin 300191, China; 4 Engineering Technology Center for Urban Soil Contamination Control and Remediation, Ministry of Ecology and Environment, Shanghai Academy of Environmental Sciences, Shanghai 200233, China; 5 Key Laboratory of Green Utilization of Critical Non-Metallic Mineral Resources, Ministry of Education, Wuhan University of Technology, Wuhan 430070, China

**Keywords:** carbon nanotubes, carbon quantum dots, sustainable agriculture, plant stress tolerance, environmental fate, plant–soil system

## Abstract

Carbon-based nanomaterials have emerged as promising tools for addressing major challenges in sustainable agriculture, including declining soil fertility, climate change, resource inefficiency, and increasing environmental pollution. Among these materials, carbon nanotubes (CNTs) and carbon quantum dots (CQDs) have attracted considerable attention because of their unique physicochemical properties and diverse interactions with plants and soil systems. This review provides a comprehensive comparative analysis of CNTs and CQDs, emphasizing how their structural characteristics govern environmental fate, plant uptake, physiological responses, and stress mitigation mechanisms. CNTs primarily function as one-dimensional nanostructures that improve soil properties, facilitate nutrient delivery, and immobilize environmental contaminants, whereas CQDs, owing to their ultrasmall size, excellent water dispersibility, and intrinsic fluorescence, actively regulate plant metabolism, photosynthesis, nutrient acquisition, and antioxidant defense. Their distinct transport pathways, rhizosphere interactions, and subcellular localization are critically evaluated alongside recent advances in synthesis, surface functionalization, and physicochemical modification. The review further summarizes current evidence regarding their roles in enhancing tolerance to heavy metal toxicity, salinity, and drought stress through modulation of reactive oxygen species scavenging, osmotic regulation, ion homeostasis, and stress-responsive signaling pathways. Potential phytotoxicity, environmental persistence, ecological risks, and green synthesis strategies are also discussed to provide a balanced assessment of their agricultural applications. Emerging opportunities for synergistic CNT–CQD composite systems are discussed, together with major knowledge gaps and future research priorities, including machine learning-assisted nanomaterial design, multi-omics characterization, long-term field validation, and life cycle-based risk assessment. This review establishes a conditional, context-dependent structure–behavior–function conceptual framework that provides theoretical guidance for the rational design and safe implementation of carbon nanomaterials in next-generation sustainable agriculture.

## 1. Introduction

Global agriculture faces unprecedented, multifaceted pressures. The world population is projected to reach 9.7 billion by 2050, driving a ~50% increase in food demand relative to current levels [1]. Concurrently, intensifying climate change-induced extreme weather events, worsening freshwater scarcity, shrinking arable land, and pervasive soil degradation and contamination collectively pose systemic threats to global food security [2,3,4]. Conventional agricultural technologies—including increased fertilizer application, pesticide spraying, and traditional breeding—are proving increasingly inadequate against these compounded challenges. Nitrogen use efficiency from fertilizers stagnates at ~40%, while excess application causes eutrophication and greenhouse gas emissions. Pesticide delivery efficiency remains below 0.1%, resulting in substantial off-target environmental contamination [5,6]. Consequently, transformative technologies enabling “increased yield with reduced environmental footprint” have become a central pursuit in agricultural science [7,8,9].

Nanotechnology offers novel pathways to address resource inefficiency and environmental risks in agriculture [10,11]. When materials are reduced to the nanoscale (1–100 nm), enhanced specific surface area, surface atom ratio, and quantum confinement effects confer properties distinct from their bulk counterparts [12]. These include superior reactivity, adsorption capacity, and optical characteristics, aligning well with agricultural demands such as precision loading, controlled release, pollutant immobilization, and early plant diagnostics. This synergy has spurred the rapidly evolving frontier of “nanoagriculture [13,14,15].”

Within the diverse nanomaterial portfolio for agriculture, carbon-based nanomaterials (CNMs) attract significant attention due to their unique carbonaceous chemistry, tunable surface functionality, and relatively low metal toxicity risks [16]. Within the carbon-based nanomaterial family, both graphene and graphene oxide (GO) are two-dimensional (2D) materials that possess outstanding mechanical strength, high specific surface area, and strong adsorption capacity, and have been reported to modulate seed germination, root development, and stress tolerance in a range of crop species. Nevertheless, pristine graphene is difficult to disperse stably in aqueous media. This is mainly due to its pronounced hydrophobicity and π–π stacking [17]. In contrast, GO has a certain degree of hydrophilicity. This hydrophilicity comes from its surface oxygen-containing functional groups [18]. However, GO remains prone to aggregation in complex media. Such media include those of high ionic strength. Moreover, both materials share large lateral dimensions. These dimensions collectively constrain their effective translocation within plant tissues. Concurrently, the long-term environmental and biological safety of graphene-based materials remains a subject of extensive debate [19]. Nanodiamonds, a zero-dimensional (0D) carbon nanomaterial, can exhibit high biocompatibility and photostable emission following specific doping treatments; however, their prohibitive fabrication cost, together with the paucity of data on their agricultural fate and effects, has thus far restricted their exploration in agronomic contexts [20]. Fullerenes, another archetypal zero-dimensional (0D) carbon cage structure, readily form nano-aggregates with heterogeneous physicochemical properties in aqueous media because of their strong hydrophobicity, and this uncontrollability of environmental behavior limits their practical deployment in soil–plant systems [21]. Against this backdrop, CNTs and CQDs stand out as metal-free carbon nanostructures: both benefit from mature, scalable synthetic routes and extensively documented plant–soil interactions and, most critically, they exhibit complementary dimensional characteristics that render them archetypal candidates for a systematic structure–behavior–function comparison. Carbon nanotubes (CNTs) and carbon quantum dots (CQDs)—representing archetypal one-dimensional tubular and zero-dimensional quasi-spherical structures, respectively—differ fundamentally in dimensionality, morphology, surface chemistry, and electronic structure. In this review, “CQDs” is used as the unified term for carbon-based quantum dots, including undoped CQDs, heteroatom-doped CQDs (e.g., N-CQDs), nutrient-loaded CQDs, and CQD hybrids; the terms “CDs” and “CNDs” appearing in cited works are treated as synonyms of CQDs. These differences dictate their distinct interactions with the soil–plant system. CNTs typically act as “physical scaffolds” and “external barriers” via “physical aggregation-persistent retention,” whereas CQDs mediate “chemical signaling” and “internal signal activation” through “chemical dispersion-biodegradable migration [22,23].” Despite a rapid growth in literature on both materials, existing reviews predominantly focus on single-material systems. Systematic, side-by-side comparisons of their environmental behavior and plant effects within a unified framework are lacking, limiting understanding of their functional complementarity and synergistic potential [24,25].

This review establishes a “structure–behavior–function” causal chain analysis framework. We systematically compared CNTs and CQDs across four dimensions: synthesis and physicochemical properties; environmental fate within the soil–plant continuum; plant growth promotion and nutrient use efficiency; and regulation of abiotic stress tolerance. Furthermore, we evaluated their environmental safety, risk assessment, and synergistic application prospects. We caution that many published CNT-vs.-CQD comparisons are confounded by differences in surface charge, oxidation state, residual catalysts, dispersants, dopants, hydrodynamic size, aggregation state, crop species, growth medium, and exposure regime. The dimensionality-based contrast below should therefore be read as a heuristic framework, not a causal attribution to 1D vs. 0D per se. This review aims to provide a theoretical foundation and roadmap for the rational design and safe deployment of nanotechnology in sustainable agriculture. The following schematic (Figure 1) maps our structure–behavior–function framework onto the practical workflow of CNT and CQD deployment in agroecosystems.

### Literature Search and Evidence Grading Strategy

To ensure a comprehensive and transparent literature coverage, we performed a systematic search in Web of Science Core Collection and Scopus (January 2000–August 2026) using the following keyword combinations: (“carbon nanotube” OR “CNT” OR “single-walled carbon nanotube” OR “multi-walled carbon nanotube”) AND (“carbon quantum dot” OR “carbon dot” OR “carbon nanodot” OR “CQD”) AND (“plant” OR “crop” OR “soil” OR “agricultur” OR “stress*” OR “germination” OR “photosynthesis”).

To visualize the thematic landscape and temporal evolution of the retrieved literature, we conducted a keyword co-occurrence analysis using VOSviewer (version 1.6.20). The network map (Figure 2a) reveals four major clusters: (i) carbon nanotube–soil–plant interactions; (ii) carbon quantum dots–stress tolerance–antioxidant defense; (iii) nanomaterial toxicity and environmental fate; and (iv) synergistic applications and sensing. The temporal overlay (Figure 2b) shows a clear shift from early toxicity-focused studies (2010–2015) toward application-oriented research on stress mitigation and sustainable agriculture (2018–2025). These bibliometric results guid the structure of this review and support the selection of representative studies for each section.

## 2. Synthesis, Functionalization, and Physicochemical Properties

### 2.1. Synthetic Strategies

Carbon nanotube synthesis centers on high-temperature gas-phase deposition, while carbon quantum dot synthesis primarily employs liquid-phase chemical routes. This fundamental divergence shapes their size distribution, crystallinity, and surface chemistry [26,27].

Single-walled carbon nanotubes (SWCNTs) and multi-walled carbon nanotubes (MWCNTs) are mainly synthesized via arc discharge, laser ablation, and chemical vapor deposition (CVD) [28,29]. Among these, CVD is the most prevalent due to its superior controllability and higher yield [30]. During CVD, hydrocarbon gases (e.g., methane, ethylene, acetylene) decompose thermally at 600–1000 °C over transition metal catalysts (Fe, Co, Ni, or alloys), catalyzing the growth of tubular carbon structures [31]. Catalyst nanoparticle size and composition critically determine CNT diameter and chirality; for instance, smaller Fe nanoparticles favor SWCNT formation, whereas larger particles direct MWCNT growth [29]. CVD-synthesized CNTs inevitably contain impurities like amorphous carbon, residual metal catalysts, and carbon shells, necessitating purification via acid washing (e.g., HNO_3_/H_2_SO_4_ mixtures) and high-temperature annealing to achieve high purity [31]. Arc discharge and laser ablation produce CNTs with fewer structural defects but are costlier and lower-yielding, limiting them primarily to fundamental research [28,32].

CQD synthesis strategies fall into “top-down” and “bottom-up” categories [33,34]. Top-down routes fragment macroscopic carbon precursors (graphite, carbon fibers, activated carbon) into nanoscale dots via arc discharge, laser ablation, or electrochemical oxidation, often yielding products rich in surface oxygen-containing groups [35]. Bottom-up approaches involve carbonizing and nucleating small organic molecules (citric acid, glucose, urea, etc.) [36]. Hydrothermal/solvothermal methods and microwave-assisted synthesis are common [37]. Hydrothermal synthesis, particularly using citric acid and ethylenediamine at 180–200 °C for 4–10 h, is widely adopted in laboratories for producing nitrogen-doped CQDs (N-CQDs) with high fluorescence quantum yields [38]. Microwave methods drastically reduce reaction time but often result in broader size distributions, requiring post-synthesis dialysis or column chromatography for separation [39].

Synthetically, CNT production relies heavily on high temperatures (>600 °C) and metal catalysts, demanding rigorous purification [40]. In contrast, CQDs form under milder conditions (150–250 °C) without metal catalysts, simplifying purification. A key implication is the potential for metal residues in CNTs, posing additional toxicity risks upon environmental release, whereas CQDs carry negligible metal contamination risks. Moreover, CNT CVD synthesis requires inert gas protection and precise flow control, presenting higher equipment barriers. Conversely, CQD hydrothermal synthesis is accessible under conventional laboratory settings, facilitating scalable production and facile one-step functionalization [41,42].

### 2.2. Surface Functionalization and Chemical Tunability

Surface chemistry dictates the dispersibility, biocompatibility, and plant interface interactions of CNMs [43,44,45]. Functionalization strategies differ markedly: CNTs primarily utilize covalent and non-covalent modifications, while CQDs rely on in-situ doping during synthesis and surface passivation [46].

Pristine CNTs are highly hydrophobic and prone to agglomeration in aqueous media, hindering their mobility and bioavailability in soil–plant systems [47]. Covalent functionalization introduces hydrophilic oxygen-containing groups (carboxyl -COOH, hydroxyl -OH, carbonyl -C=O) onto CNT sidewalls and ends, commonly via acid treatment [48,49]. While effective for improving dispersibility, excessive oxidation damages the sp^2^ carbon skeleton, compromising mechanical strength and electrical conductivity. Balancing functionality and structural integrity is crucial [50]. Non-covalent functionalization employs π–π stacking, hydrophobic interactions, or electrostatic adsorption to wrap surfactants, polymers, or biomolecules (DNA, proteins, humic acid) around CNTs. This preserves the sp^2^ structure while enhancing dispersion [51]. For example, humic acid coating improves colloidal stability via electrostatic repulsion and steric hindrance, simultaneously mimicking natural organic matter interactions in field soils, offering a realistic model for studying CNT behavior [52].

CQDs offer greater flexibility and tunability in surface engineering [46]. Firstly, synthesis and functionalization can be integrated: selecting specific precursor combinations allows in-situ doping of heteroatoms (N, S, P, B) into the carbon core alongside surface group modulation [53,54]. Secondly, post-synthetic modification via amidation, esterification, or silanization enables conjugation of targeting moieties (antibodies, aptamers, folic acid) [55]. Crucially, CQDs inherently possess abundant surface carboxyl and hydroxyl groups, granting excellent water dispersibility without mandatory pre-treatment—a significant advantage over CNTs for environmental applications [56].

Functionalization of CNTs is largely “remedial”—transforming an intrinsically hydrophobic surface into a hydrophilic one to overcome agglomeration tendencies. In contrast, CQD functionalization is “enhancive”—building upon an already hydrophilic surface to fine-tune optical properties, chemical reactivity, and bio-recognition capabilities [57,58]. This fundamental difference implies that CNT behavior in soil is dictated by the degree of functionalization and coating stability, whereas CQD behavior is governed more by surface ligand density and charge state [25].

### 2.3. Comparative Overview of the Structural and Optical Properties

CNTs and CQDs present stark contrasts in dimensionality, crystallinity, and optical properties, summarized in Table 1 [33,59].

CNTs are 1D tubular structures formed by rolling graphene sheets composed of sp^2^-hybridized carbon atoms, exhibiting aspect ratios of 10^3^–10^6^ [60]. SWCNTs are seamless cylinders of single-layer graphene; their chirality vector (n, m) determines electronic properties: metallic if n−m is a multiple of three, otherwise semiconducting [61]. MWCNTs consist of multiple concentric graphene tubes with an interlayer spacing (~0.34 nm) similar to graphite [62]. CQDs are 0D quasi-spherical nanoparticles with a carbon core comprising a mixture of sp^2^/sp^3^ hybridized carbon, typically 2–8 nm in size [37]. The CQD core can be amorphous carbon, nanocrystalline graphite, or diamond-like carbon, generally possessing lower crystallinity than CNTs. This lower crystallinity, combined with abundant surface defects and edge states, underpins CQDs’ unique optical properties [63]. CNTs exhibit very weak intrinsic fluorescence, primarily from defect-bound exciton recombination, with quantum yields usually <1% [64]. Conversely, CQDs display rich photoluminescence (PL), notably excitation wavelength-dependent emission—where emission peaks redshift with increasing excitation wavelength—attributed to diverse emissive sites on the surface and quantum confinement effects [65,66,67].

CQD quantum yields can be significantly enhanced via surface passivation and heteroatom doping [46]. Additionally, CQDs exhibit upconversion PL, emitting visible light upon near-infrared excitation, offering potential for plant light manipulation and bioimaging [68,69,70]. CNTs’ exceptional electrical conductivity is a structural asset; metallic SWCNTs rival copper, while semiconducting SWCNTs surpass silicon in carrier mobility [71]. However, achieving precise chirality control remains challenging [29]. CQDs behave as semiconductors with weaker conductivity due to discontinuous sp^2^ domains and abundant surface defects [72]. In soil–plant systems, these conductivity differences may influence interactions with root cell membrane potentials and migration within soil electrochemical gradients.

Fundamental synthetic disparities create divergent starting points regarding impurity residues and scalability potential. Functionalization strategies—remedial for CNTs versus enhancive for CQDs—directly govern dispersibility and interfacial behavior in media. Distinct dimensionalities, crystallinities, and specific surface areas lead to contrasting transport, adsorption, and root contact modes. Markedly different optical properties delineate their respective capabilities for tracing and light-regulation applications. This structure-to-property logic forms the analytical basis for examining subsequent differences in their migration, uptake, and effects within the soil–plant continuum.

## 3. Environmental Behavior in the Soil-Plant Continuum

### 3.1. Fate and Transport in Soil Environments

The environmental fate of carbon nanomaterials in soil encompasses physical transport, chemical transformation, and biodegradation. CNTs and CQDs exhibit distinct fates due to their structural and surface chemical differences [73,74].

CNT transport in soil is severely constrained by their strong hydrophobicity and high aspect ratio [75]. They readily undergo homogeneous aggregation in aqueous phases; van der Waals forces and π–π stacking between tubes prevent stable dispersion of individual CNTs at environmentally relevant concentrations [76]. Kasel et al. demonstrated limited effective transport distances for CNTs in soil [77]. O’Carroll et al. further highlighted the significant impact of diameter: smaller-diameter MWCNTs exhibited lower mobility than larger ones [78]. Sharma et al. confirmed via soil column experiments that surface functional groups critically enhance MWCNT stability and alter their retention during transport through porous media [79]. Zhang et al. showed that even functionalized MWCNTs (f-MWCNTs) migrated poorly in natural soils, with divalent cations significantly amplifying their retention [80]. Biodegradation of CNTs is possible via peroxidases secreted by certain soil fungi and bacteria, but rates are extremely slow and heavily dependent on defect density and functionalization degree [81].

In contrast, CQDs, endowed with hydrophilic surface functionalities (carboxyl, hydroxyl, amino groups), exhibit excellent colloidal dispersibility in soil solution [37]. Their small size (2–10 nm) and quasi-spherical geometry ensure Brownian motion overcomes gravitational settling, enabling long-term suspension under low ionic strength [82]. Nevertheless, CQD transport in real soils is modulated by multiple factors. Increasing ionic strength or the presence of multivalent cations shields negative surface charges and promotes bridging by Ca^2+^, inducing gradual CQD aggregation [83]. CQDs generally migrate more readily than CNTs, attributable to three factors [84,85]: (1) Their 0D spherical structure minimizes physical straining in porous media compared with 1D tubular CNTs; (2) Abundant hydrophilic groups confer stronger electrostatic repulsion and steric hindrance; (3) Their sub-10 nm size is smaller than most soil pore throats, facilitating movement under saturated and unsaturated flow. CQDs are not entirely inert; soil organic matter like humic acid (HA) and fulvic acid (FA) binds to CQD surfaces via hydrogen bonding and hydrophobic interactions [86]. Furthermore, CQDs’ rich surface functionalities render them more susceptible to microbial metabolic utilization [87].

CNTs’ strong soil retention implies environmental risks concentrated in surface horizons and the rhizosphere, potentially causing chronic effects on soil biota upon accumulation [88]. Conversely, CQDs’ higher mobility disperses risks spatially and increases the likelihood of groundwater contamination [89]. These divergent fates provide essential context for discussing plant uptake and rhizosphere microbiome modulation.

### 3.2. Plant Uptake, Translocation, and Subcellular Localization

The ability of carbon nanomaterials to enter plants and translocate internally dictates their potential to enter food chains and exert physiological effects [90].

Accumulating evidence confirms CNT penetration into vascular tissues under specific conditions. Smirnova et al. observed MWCNT structures within root and leaf tissues of *Onobrychis arenaria* exposed to MWCNTs using transmission electron microscopy (TEM) [91]. Zhai et al. demonstrated charge-selective uptake of three types of MWCNTs in maize and soybean: positively charged MWCNT-NH_2_ and neutral p-MWCNT translocated more efficiently, while negatively charged MWCNT-COOH faced limitations crossing membranes [92]. Positively charged CNTs, despite strong electrostatic binding to cell walls, may trigger clathrin-mediated endocytosis for symplastic entry. Negatively charged CNTs predominantly accumulate in apoplastic spaces, limiting apparent translocation efficiency. CNT diameter and length critically influence uptake; diameters smaller than cell wall pores (typically 5–20 nm) facilitate passage, whereas lengths exceeding cortical cell gaps cause surface entrapment [93].

From a plant physiological perspective, nanoparticle translocation involves apoplastic and symplastic pathways. Liu et al. earlier confirmed SWCNT penetration of cell walls and membranes but noted predominant apoplastic enrichment [94]. Stampoulis et al. reported a 60% biomass reduction in zucchini after 15 days of hydroponic exposure to 1000 mg/L MWCNTs, indicating significant toxicity alongside uptake. However, lower concentrations (10–40 mg/L) promoted plant growth [90]. Khodakovskaya et al. corroborated that MWCNTs within this range significantly enhanced seed germination and growth in tomato, soybean, and maize, tripling seedling fresh weight [95]. Subcellular distribution studies reveal compartmentalization: MWCNTs accumulate in xylem and phloem cells, localizing to cytoplasm, cell walls, membranes, chloroplasts, and mitochondria. Chloroplast accumulation raises concerns about interference with photosynthetic electron transport [92].

CQDs, owing to their small size and hydrophilicity, are expected to be taken up more efficiently. They can enter roots via both apoplastic and symplastic pathways [96]. Sun et al. showed that quantum dot (QD) aggregation on root cell walls in maize seedlings is regulated by wall polysaccharide components; pectin and hemicellulose carboxyl groups interact with QD surface ligands via Ca^2+^ bridges, modulating trans-wall transport [97]. Long-distance CQD translocation primarily occurs via xylem vessels driven by transpiration stream. Studies with Ce-doped CQDs (Ce-CQDs) in wheat confirmed root uptake followed by upward translocation to leaves, accumulating in leaf veins [98]. Dong et al. further verified that Ce-CQDs absorbed by mung bean roots moved via vascular systems and apoplastic pathways to stems, intercellular spaces, stomata, and veins, concentrating in vascular tissues [99]. These findings demonstrate CQDs’ capacity for systemic distribution. Should CQDs accumulate in edible tissues, rigorous assessment of human dietary exposure risk is imperative.

CQD fluorescence enables direct subcellular tracking via confocal laser scanning microscopy (CLSM), unlike CNTs, which often require fluorescent labeling. CQDs can enter organelles including nuclei and mitochondria. Nuclear localization raises genetic toxicity concerns. Research indicates CQDs induce gene methylation changes post-uptake, with such epigenetic effects heritable to T1 generation seeds [100]. This underscores the need for systematic evaluation of CQDs’ long-term impacts on plant genome stability at agriculturally relevant concentrations.

Notably, the localization evidence cited above is qualitative and largely confined to vascular tissue; no whole-plant mass balance currently exists that quantifies the fraction of particles crossing plasma membranes, translocating to edible organs, remaining intact, being biotransformed, or excreted. Quantitative whole-plant fate studies, ideally with isotopically labeled materials, are therefore an outstanding priority. It should be emphasized that a large proportion of experimental evidence discussed throughout Section 4 and Section 5 originates from controlled laboratory-scale assays including hydroponic culture, agar media, and seedling-stage experiments, often employing doses that may not be agronomically realistic under real-field conditions. Accordingly, physiological observations derived from such lab settings should be generalized to field-scale agricultural production only with considerable caution.

### 3.3. Rhizosphere Microbiome Modulation

The rhizosphere, the most biologically active interface in the soil–plant continuum, is profoundly influenced by carbon nanomaterial accumulation and transformation [101,102,103].

MWCNTs directly promote the colonization of beneficial rhizobacteria. Studies show that 10–100 mg/L MWCNTs significantly enhance biofilm formation by the plant growth-promoting rhizobacterium (PGPR) *Bacillus subtilis* strain Tpb55. This involves increased exopolysaccharide and protein production and upregulated expression of biofilm-related genes. Enhanced biofilm formation translates to more effective root colonization, providing sustained growth promotion and disease protection [104]. This reveals CNTs’ capacity to indirectly optimize the rhizosphere microenvironment by modulating specific microbial physiology.

Conversely, CQDs tend to exert systemic effects on overall microbial community structure and function. Applying biomass-derived carbon dots as soil amendments significantly elevates soil organic matter content and nutrient cycling rates in the maize rhizosphere. Metagenomic analyses indicate CQD treatments enrich key microbial taxa involved in carbon, nitrogen, and phosphorus metabolism, such as *Sphingomonas*, *Bacillus*, and *Pseudomonas*, whose heightened activity directly stimulates plant growth [105]. Another study on nitrogen-doped carbon dots found they induced maize roots to secrete more organic acids [106]. These exudates act as chemical signals and carbon sources, further shaping a distinct rhizobacterial community, creating a positive feedback loop.

However, these modulatory effects carry potential risks. Nanomaterial dose, surface functionalization, and environmental aging critically influence ecological outcomes. Excessive or inappropriate application could inhibit non-target microbes or disrupt established ecological equilibria [107,108].

## 4. Plant Growth Promotion and Nutrient Use Efficiency

### 4.1. Seed Priming and Germination Enhancement

Seed germination represents a critical window sensitive to external cues. Nanopriming enhances germination uniformity and crop resilience by activating antioxidant defenses and upregulating aquaporin gene expression [109,110].

Khodakovskaya et al. pioneered research on CNT-mediated germination enhancement. Exposing tomato seeds to CNTs on agar media significantly increased germination rates. CNTs penetrated the tough seed coat and accumulated internally, facilitating water uptake [95]. Lahiani et al. extended these findings to barley, soybean, and maize, demonstrating that MWCNTs applied via various routes promoted germination and early growth. Raman spectroscopy and TEM confirmed CNT accumulation within seeds, while RT-PCR revealed upregulation of multiple aquaporin genes, linking CNT-enhanced water uptake to aquaporin modulation [111,112]. Importantly, priming efficacy is crop- and cultivar-specific [113]. Generally, large-seeded crops with thick coats exhibit more pronounced responses to CNT physical penetration than small-seeded, thin-coated species [111].

Unlike CNTs’ physical mechanism, CQDs primarily promote germination via surface chemical activity and phytohormone-like effects. Kou et al. treated lettuce and tomato seeds with CQDs synthesized hydrothermally from cysteine and glucose, observing accelerated germination, enhanced root and hypocotyl elongation, and upregulated aquaporin expression [114]. Similar to CNTs, CQDs improved seed water uptake; however, their promotive effects occurred at lower concentrations, exhibited flatter dose–response curves, and possessed wider safety margins [115,116]. Furthermore, CQD surface functionalities can mimic phytohormones, particularly auxins. Surface carboxyl groups may interact with auxin receptors (e.g., TIR1/AFB), although direct biophysical evidence for such binding remains lacking (see Section 8 for discussion) [117,118]. CQDs also induce elevated α-amylase activity, accelerating stored starch degradation and soluble sugar accumulation to fuel embryo growth [119]. Yu et al. reported that nitrogen–phosphorus co-doped CQDs promoted water spinach growth via foliar and root application, linked to improved photosynthetic performance and elevated total phenolic content, anthocyanins, and flavonoids [120].

### 4.2. Root System Architecture and Nutrient Acquisition

Root system architecture (RSA)—encompassing primary root length, lateral root density, root hair abundance, and root–shoot ratio—dictates nutrient acquisition efficiency [121,122,123,124].

CNT effects on root development are concentration-dependent and crop-specific. Lahiani et al. confirmed MWCNT promotion of germination and early growth in barley, soybean, and maize, linked to testa penetration and aquaporin upregulation enhancing water uptake [111]. However, high MWCNT concentrations (1000–2000 mg/L) significantly inhibited root and shoot elongation in red spinach and lettuce, with varying sensitivity: red spinach and lettuce were most sensitive, while chili, okra, and soybean showed marginal or no toxicity [121]. Tan et al. further observed MWCNT inhibition of root elongation in tomato and lettuce, stemming from “interaction with the root surface” rather than internalization [125]. CNTs also influence nutrient acquisition by modulating rhizosphere nutrient availability. Wani et al. noted that CNTs’ high specific surface area adsorbs NH_4_^+^, NO_3_^−^, PO_4_^3−^, and K^+^ ions from soil solution, creating a slow-release nutrient reservoir near roots, prolonging nutrient supply [113].

CQDs robustly promote root development across a broad effective concentration range. Kou et al. reported that CQDs significantly enhanced root elongation and lateral root formation in lettuce and tomato, correlating with upregulated aquaporin genes and improved mineral nutrient uptake [114]. Tan et al. studied N-CQD effects on lettuce yield and quality, finding that 100–200 mg/L N-CQDs increased biomass by 41.70%, alongside elevated chlorophyll a content, electron transport rate (ETR), and photosystem II quantum efficiency (ΦII). Rubisco activity surged by 60.61% [126]. These effects suggest a positive feedback loop where CQD-enhanced root growth fuels improved aboveground photosynthesis. CQDs themselves serve as micronutrient carriers. Alikhani et al. developed a carbon dot-zinc complex as a slow-release Zn fertilizer, significantly boosting grain Zn concentration and growth parameters in wheat. Moreover, CQDs upregulate genes encoding nutrient transporters. Studies indicate CQDs elevate transcript levels of nitrate transporter (NRT) and potassium channel (AKT) genes, enhancing active nutrient uptake [127,128].

Under stress, CQD modulation of RSA is particularly notable. Wang et al. examined nitrogen-doped and polyacrylic acid-modified carbon dots (PNDs) on drought-stressed maize. Foliar PND application reduced ROS accumulation, enhanced net photosynthetic rate by 206.8%, and promoted abscisic acid (ABA) and proline accumulation in roots, strengthening water uptake capacity. Dynamic monitoring revealed ABA and proline synthesis initiated in leaves, followed by long-distance transport to roots [129]. This unveils a novel CQD mechanism regulating RSA via shoot-to-root signaling.

While CNTs predominantly adsorb to root surfaces with limited translocation, CQDs’ metabolic lability and systemic distribution make them suitable for repeated application strategies in precision agriculture.

### 4.3. Photosynthesis and Carbon Metabolism

Photosynthesis drives biomass accumulation [130,131,132]. CNT effects on photosynthesis are complex and context-dependent. On one hand, CNTs can elevate leaf pigment content. Gonzalez-Garcia et al. reported increased total chlorophyll and enhanced activities of ascorbate peroxidase (APX), glutathione peroxidase (GPX), and phenylalanine ammonia-lyase (PAL) in tomato seedlings treated with CNTs or graphene [133]. These responses might stem from moderate oxidative stress triggering defensive upregulation of pigment biosynthesis genes. On the other hand, CNT impacts on the photosynthetic electron transport chain (ETC) are dual-edged. Lambreva et al. employed steady-state and time-resolved fluorescence spectroscopy to investigate SWCNT effects on isolated thylakoid membranes and PSII-enriched membranes. SWCNTs induced chlorophyll fluorescence quenching and shortened average fluorescence lifetime but showed no direct energy transfer to pigment–protein complexes. Instead, SWCNTs appeared to intercept electrons on the PSII acceptor side. This “electron leakage” could exacerbate photoinhibition under high light [134].

In contrast, CQD mechanisms for enhancing photosynthesis are more defined, capitalizing on their unique photophysical properties. Four key mechanisms operate: First, light-harvesting enhancement. CQDs exhibit broad UV-Vis absorption and strong fluorescence [135]. Crucially, some possess upconversion luminescence (UCPL), absorbing near-infrared (NIR) light and emitting visible photons. This “antenna effect” provides chloroplasts with supplemental photosynthetically active radiation (PAR), theoretically boosting light use efficiency (LUE) [136]. Upconversion photoluminescence is an anti-Stokes process in which a material absorbs two or more low-energy near-infrared (NIR) photons and emits one higher-energy visible photon. In CQDs, this process typically arises from multi-photon absorption or energy transfer involving surface states, defect states, or dopant-induced energy levels. The absorbed NIR photons excite electrons to higher energy states, which then relax radiatively to emit visible light. This property enables CQDs to convert NIR light, which chloroplasts cannot directly utilize, into photosynthetically active radiation (PAR), thereby potentially supplementing light availability for photosynthesis [137]. Wang et al. showed functionalized carbon dots target chloroplasts in vivo, functioning as light converters [129]. Second, ETC acceleration. CQDs act as electron donors or shuttles within the ETC, expediting electron flow from PSII to PSI. Tan et al. documented a 38.61% increase in ETR and a 31.24% rise in ΦII in N-CQD-treated lettuce leaves [126]. Third, increased activity of carbon-fixing enzymes. Ribulose-1,5-bisphosphate carboxylase/oxygenase (Rubisco) is the rate-limiting enzyme for CO_2_ fixation [138]. Tan et al. found N-CQDs boosted Rubisco activity by 60.61%, alongside increased glutamine synthetase activity, indicating concurrent enhancement of nitrogen assimilation [126]. Yu et al. further confirmed that heteroatom doping amplifies CQD regulatory capacity on photosynthetic carbon metabolism [120]. Fourth, accumulation of carbon metabolites. CQD treatment elevates leaf soluble sugars, starch, and organic acids, confirming that improved carbon fixation translates into tangible metabolite pools [114,129]. These compounds fuel growth and serve as osmolytes (e.g., proline, soluble sugars), bolstering stress tolerance [139].

CNT photosynthetic modulation is best characterized as “passive adjustment”—indirectly influencing photosynthesis by altering the microenvironment of photosynthetic machinery. Conversely, CQD effects constitute “active enhancement”—directly participating in and optimizing photosynthetic processes via photophysical and photochemical mechanisms.

## 5. Regulation of Abiotic Stress Tolerance

### 5.1. Heavy Metal Stress

Heavy metal contamination is a pervasive stressor in agricultural soils [140,141,142]. CNTs alleviate heavy metal stress primarily through their exceptional adsorption capacity. With specific surface areas reaching 150–1315 m^2^/g and surfaces enriched with π–electrons, oxygen groups, and topological defects, CNTs exhibit high affinity for divalent cations (Cd^2+^, Pb^2+^, Cu^2+^, Zn^2+^) [143]. Adsorption mechanisms encompass electrostatic attraction, ion exchange, π–cation interactions, and surface complexation [144]. Functionalized MWCNTs can achieve metal adsorption efficiencies up to 20-fold higher than pristine counterparts [145]. However, limitations exist. CNT agglomeration in soil drastically reduces their effective surface area [146]. Furthermore, the “Trojan horse effect”—where metals adsorbed to CNTs enter plants along with the CNTs—may inadvertently increase internal metal exposure [147]. Such limitations necessitate cautious evaluation of long-term ecological risks when deploying CNTs as adsorbents.

CQDs mitigate heavy metal stress predominantly by activating endogenous plant defense and detoxification pathways. Xiao et al. investigated CQD effects on Cd-stressed wheat seedlings. Hydroponic CQD addition significantly alleviated growth inhibition, reducing Cd content in roots and leaves by 33.1–57.7%. Mechanistically, CQDs restored the activities of antioxidant enzymes (APX, CAT, POD) perturbed by Cd and modulated genes related to glutathione synthesis (ATPS, APR, SiR, CS, GS). Specifically, Cd alone suppressed ATPS expression by 36.9%, while CQDs reversed this suppression [148]. Chen et al. demonstrated that amphoteric N-CQDs mitigated Cd stress in *Arabidopsis thaliana*, elevating CAT and POD activities to 2.73-fold and 1.45-fold of controls, respectively [149]. Cheng et al. compared foliar CQD sprays on Cd-stressed rice, observing reductions in root and shoot Cd accumulation by 31.51% and 17.93%. Transcriptomics and mineral analysis revealed CQDs inhibited Cd uptake by downregulating *OsNramp1* while enhancing competitive binding of Fe/Mn to *OsNramp5*. Concurrently, CQDs downregulated *OsHMA2* and upregulated *OsHMA3*, impeding translocation. Moreover, CQDs promoted root development, enhancing Cd sequestration by root-associated organic matter and iron/manganese oxides, thereby reducing Cd bioavailability [150].

### 5.2. Salinity–Alkalinity Stress

Soil salinization suppresses plant growth through osmotic stress, ion toxicity, and oxidative damage [151,152,153].

Zhao et al. studied MWCNT effects on salt-stressed rapeseed seedlings, finding MWCNTs alleviated growth inhibition [154]. Xu et al. reported that MWCNTs optimized ion homeostasis in salt-stressed rice seedlings by regulating Na^+^/K^+^ selective transport, reducing cytosolic Na^+^ accumulation while maintaining higher K^+^ levels [155]. However, CNT efficacy is concentration-dependent. Luo et al. showed that 20 mg/L MWCNTs promoted proline and K^+^ accumulation in salt-stressed maize leaves, but this benefit diminished at 50 mg/L, coinciding with elevated leaf Na^+^ content and Na^+^/K^+^ ratios. This suggests low-dose CNTs confer protection, while higher doses may exacerbate salinity stress [156].

CQDs alleviate salt stress through multiple interconnected pathways: (1) Accumulation of osmolytes (proline, glycine betaine, soluble sugars); (2) Activation of antioxidant systems (SOD, CAT, APX, GPX, GR); (3) Reprogramming ion homeostasis (enhanced Na^+^ efflux, improved K^+^ retention); (4) Photosynthetic protection (maintenance of chlorophyll content, preservation of PSII photochemistry) [25]. Biomass-derived CQDs effectively scavenge hydroxyl radicals (·OH), mitigating oxidative damage in mung bean under hydroponic and soil-based salinity [157]. Nitrogen-doped CQDs significantly enhanced antioxidant enzyme activities (SOD, CAT, APX) and reduced malondialdehyde (MDA, a lipid peroxidation marker) content in tomato and mung bean, protecting membrane integrity [158]. Glycosylated CQDs stimulated greater accumulation of proline and soluble sugars in salt-stressed mung bean, improving cellular osmotic potential [159].

### 5.3. Drought Stress

Drought stress poses a systemic threat to agricultural production [160,161,162]. CNT mitigation of drought stress leverages their physicochemical modulation of soil–plant water relations. Their high surface area and nanopores adsorb and retain soil moisture, reducing evaporation rates [163]. Exogenous CNT application enhances plant water status; studies report improved relative water content and stabilized water potential in drought-stressed chili (*Capsicum annuum* L.), maintaining cellular turgor and basal metabolism [164]. This partially stems from CNT-mediated stomatal regulation. In boreal shrubs like *Shepherdia canadensis* and *Cornus sericea*, carboxylated MWCNTs altered stomatal conductance dynamics under drought, prompting earlier or more efficient closure to minimize transpirational water loss [165]. CNTs also protect the photosynthetic apparatus. In peanut (*Arachis hypogaea*), MWCNTs increased chlorophyll a, b, and total chlorophyll content, and enhanced the maximum quantum efficiency of PSII (Fv/Fm), indicating protection of the electron transport chain [166]. Confronting drought-induced oxidative damage, CNTs activate robust antioxidant defenses. Drought elevates reactive oxygen species (ROS), causing lipid peroxidation. Studies show carbon nanodot treatment boosts activities of key antioxidants (SOD, POD, CAT) in maize and promotes synthesis of non-enzymatic antioxidants like phenolic acids, effectively scavenging ROS and lowering MDA [167]. In chili, CNTs also induced massive accumulation of osmolytes like proline and soluble sugars, aiding water retention and ROS scavenging [164].

Research on CQD-mediated drought stress mitigation has yielded significant advances across multiple levels. A core mechanism is their potent ROS-scavenging capacity. Blue-emitting antioxidant CQDs (B-CQDs) synthesized from citric acid and ascorbic acid enhanced drought resistance in pea by effectively neutralizing ROS, preserving membrane integrity and organelle function [168]. Regarding water relations, CQDs optimize water use efficiency (WUE) multifariously. Firstly, they promote osmolyte accumulation (proline, soluble sugars, soluble proteins), lowering osmotic potential to aid water uptake under low soil water potential, thus maintaining leaf relative water content (RWC) and turgor [169]. Secondly, CQDs modulate abscisic acid (ABA) signaling. By regulating ABA-related genes (e.g., NCED, PYR/PYL receptors, PP2C phosphatases, SnRK2 kinases), CQDs optimize stomatal behavior, achieving efficient water use without excessively compromising photosynthesis [129]. Some studies note improved RSA (increased root length, surface area, root–shoot ratio), expanding water acquisition. At the molecular level, CQDs trigger expression changes in drought-responsive genes [170]. In soybean, CQDs promoted amino acid biosynthesis and enhanced overall physiological efficiency via nitrogen metabolism gene regulation [171]. Remarkably, in sweet potato, red-emissive CQDs activated melatonin biosynthesis genes via their photoluminescence. This melatonin upregulation subsequently enhanced potassium (K^+^) homeostasis gene expression through melatonin signaling, fortifying ion balance and drought resilience [172].

### 5.4. Summary: Commonalities and Divergences in Stress Mitigation Mechanisms from a Multi-Omics Perspective

Synthesizing the responses to heavy metals, salinity, and drought, multi-omics insights reveal shared and distinct mechanisms between CNTs and CQDs. Commonality resides in core plant defense pathways: both activate SOD-, CAT-, POD-based antioxidant systems to scavenge excess ROS and mitigate membrane lipid peroxidation. Both induce accumulation of osmolytes (proline, soluble sugars), maintaining cellular osmotic balance and photosynthetic stability. Furthermore, both regulate stress-responsive genes and metabolic pathways, establishing conserved physiological and molecular resilience frameworks [173].

Material-specific mechanistic divergences define their functional niches. CNTs primarily exert “soil-end” modulation supplemented by “plant-end” effects. Leveraging high surface area and unique nanostructure, they immobilize soil heavy metals and conserve soil moisture, reducing stress intensity at its source. However, their efficacy is constrained by concentration and soil conditions; agglomeration reduces effectiveness, high concentration causes toxicity, and metal mobilization risks exist. Plant-level molecular regulation is often a passive physiological response with weaker pathway specificity. Conversely, CQDs drive “plant-end” precision molecular regulation with negligible direct soil remediation capacity. They actively target multiple key pathways: heavy metal detoxification, ion homeostasis, ABA signaling, melatonin synthesis, and nitrogen metabolism. By remodeling gene expression and metabolic networks, CQDs systematically unlock endogenous stress resilience, offering broader regulatory scope, superior targeting, and greater proactive control.

In essence, CNTs excel in ameliorating the stress environment and passivating stressors in situ, while CQDs specialize in optimizing intrinsic plant defense circuitry. These mechanistic insights lay the groundwork for deeper multi-omics dissection and tailored applications against diverse agricultural abiotic stresses. To provide a more intuitive understanding of this dual-action strategy, Figure 3 illustrates the simplified mechanisms by which CNTs and CQDs counteract stresses.

## 6. Risk Assessment and Sustainability Considerations of Carbon Nanomaterials in Agricultural Applications

### 6.1. Hazard Assessment of Phytotoxicity and Ecotoxicity 

Evaluating Nanomaterial Is the Primary Gatekeeper for Safe Agricultural Applications [174,175,176].

#### 6.1.1. Phytotoxicity

CNT phytotoxicity has been systematically investigated across multiple species, primarily involving physical damage, oxidative stress, and nutritional interference. Their high aspect ratio and rigidity enable penetration of cell walls and membranes. This causes mechanical injury. For example, SWCNTs can traverse the mesophyll cell wall of *Arabidopsis* and induce apoptosis [177]. Oxidative stress constitutes another core mechanism. CNTs induce intracellular ROS overproduction, leading to lipid peroxidation, protein carbonylation, and DNA damage [178]. Moreover, CNTs can also induce toxicity through epigenetic modification. For example, they trigger global hyperacetylation of histone H3 in rice root apical meristems [179]. The phytotoxicity of CNTs typically follows a hormetic dose–response curve. Low concentrations may stimulate growth, whereas high concentrations cause severe toxicity. This necessitates precise dose control in agricultural applications [180].

CQDs are generally considered biocompatible. Reports of their phytotoxicity remain scarce. However, emerging studies have revealed potential toxicity under specific conditions [25]. For example, CQDs at high concentrations can inhibit primary-root meristem growth in Arabidopsis. They also accumulate within the nuclei of meristematic cells. Meanwhile, they downregulate auxin biosynthesis genes and disrupt the expression of genes involved in DNA repair and cell-cycle regulation [181].

#### 6.1.2. Impacts on Non-Target Organisms

Beyond plants, CNTs and CQDs may also affect non-target organisms in soil ecosystems. Soil microorganisms are essential to soil health and nutrient cycling. Both material classes can modulate rhizosphere microbial communities. For example, MWCNTs promote biofilm formation by beneficial rhizobacteria, whereas CQDs enrich key microbial taxa involved in carbon, nitrogen, and phosphorus metabolism [182,183]. However, excessive or inappropriate application may inhibit non-target microbes and disturb existing ecological equilibria [184]. Effects on soil fauna, such as earthworms and nematodes, also warrant attention. The physicochemical properties and reactivity of CNTs and CQDs may adversely affect their physiology and behavior, although relevant studies remain limited.

### 6.2. Environmental Exposure, Persistence, and Transformation

The exposure routes, persistence, and transformation of CNTs and CQDs in soil environments are critical to ecological risk assessment. These factors collectively determine their environmental fate.

#### 6.2.1. Environmental Exposure

CNTs and CQDs can enter agricultural soils through direct application or indirect pathways. Direct routes include soil amendments and nano-fertilizers. An indirect route is wastewater irrigation. Once in soil, their concentration in the soil solution, adsorption onto soil particles, and accumulation in the rhizosphere determine the actual exposure level of organisms. The strong hydrophobicity and high aspect ratio of CNTs restrict their transport in soil [185]. They therefore tend to accumulate in surface horizons and the rhizosphere. In contrast, CQDs exhibit good colloidal dispersibility in the soil solution owing to their hydrophilic surface functionalities and nanoscale dimensions [185]. Their mobility is consequently higher. This increases the likelihood of groundwater contamination.

#### 6.2.2. Persistence and Transformation

The persistence of CNTs and CQDs in the environment determines their long-term risk. The biodegradation of CNTs is extremely slow. It also depends strongly on defect density and the degree of functionalization. This implies that CNTs may persist in soil for extended periods. Over time, they may undergo aggregation, fragmentation, or degradation of surface coatings. Such processes can generate secondary micro-/nano-carbon particles [186]. The environmental behavior and toxicity of these particles remain poorly understood. CQDs possess richer surface functionalities. They are therefore more susceptible to microbial metabolic utilization [187]. Nevertheless, their mineralization rates under field conditions and the toxicity of their metabolites still require in-depth investigation. Soil organic matter, such as humic acid, and ionic strength also modulate the aggregation and stability of CNMs. These factors in turn alter their environmental fate and bioavailability.

### 6.3. Food-Chain Transfer and Chronic Risk

Uptake, translocation, and subcellular localization of CNTs and CQDs in planta are key steps for their entry into the food chain.

#### 6.3.1. Food-Chain Transfer

Under specific conditions, CNTs can penetrate the vascular tissue of plants. They then accumulate in xylem and phloem cells, the cytoplasm, cell walls, membranes, chloroplasts, and mitochondria. Owing to their small size and hydrophilicity, CQDs are considered to have higher uptake efficiency [92,188,189]. They undergo long-distance translocation to leaves via the xylem. They can even enter the nucleus and mitochondria. If CNMs, particularly CQDs, accumulate in edible tissues, they pose a potential risk of human dietary exposure. This requires rigorous assessment [190]. At present, studies on the trophic transfer and biomagnification of CNMs along food chains, from plants to herbivores and then to carnivores, are very limited. This remains a critical knowledge gap.

#### 6.3.2. Chronic Risk

Long-term accumulation of CNTs and CQDs in soil may cause chronic toxicity to soil ecosystems and plants. The strong retention of CNTs in soil implies that environmental risks concentrate in surface horizons and the rhizosphere. Such long-term accumulation may exert chronic effects on soil biological communities. CQDs exhibit higher mobility. Nevertheless, their long-term environmental behavior and potential chronic effects still require in-depth evaluation. These effects include long-term impacts on plant genome stability. Repeated application of nanomaterials may progressively increase their concentrations in soil. This elevates the chronic risk [185].

### 6.4. Occupational Exposure

During the production, formulation, and agricultural application of nanomaterials, operators may be exposed to CNMs through inhalation, dermal contact, or accidental ingestion. The nanoscale dimensions of CNMs confer high biological activity and potential health risks. For example, the fibrous structure of CNTs may induce asbestos-like pulmonary inflammatory responses [191]. Therefore, appropriate protective measures must be implemented throughout the life cycle of CNMs. Strict operating procedures should also be established. These actions will minimize the risk of occupational exposure. Table 2 below integrates data from multiple studies. It aims to elucidate the dose–response relationships and potential risks of different carbon nanomaterials in specific agricultural scenarios.

### 6.5. Green Synthesis, Sustainable Design, and Life Cycle Assessment

The sustainable application of CNTs and CQDs in agriculture requires source-level control. Their environmental impacts must therefore be minimized at the source.

#### 6.5.1. Green Synthesis and Sustainable Design

Conventional synthesis routes for CNTs and CQDs often rely on fossil-derived carbon feedstocks. They also involve high energy consumption and toxic catalysts or solvents. These features collectively result in a substantial environmental footprint [198]. Green synthesis and sustainable design principles aim to minimize such impacts. These principles employ benign precursors, optimize processing, and engineer material surfaces. Surface engineering enhances degradability and reduces toxicity. For example, using agricultural or forestry waste as carbon precursors to synthesize CNTs and CQDs can markedly reduce the environmental burden. Utilizing agricultural/forestry wastes (coconut shells, rice husks, bagasse) as carbon precursors represents progress in green CNT synthesis. However, current biomass-derived CNTs suffer from low yields and high defect densities, rendering them impractical for large-scale agriculture presently [199]. For CQDs, hydrothermal synthesis is inherently greener. Numerous natural biomasses—citrus juice [200], water hyacinth [201], castor seeds [202], cactus, agave extracts [203]—serve as sole precursors, reacting at 180–250 °C to yield high-quantum-yield CQDs without toxic additives [204].

#### 6.5.2. Life Cycle Assessment Framework and Techno-Economic Considerations

Life Cycle Assessment (LCA) is indispensable for validating genuine sustainability, evaluating impacts from raw material extraction, production, use, to end-of-life disposal, avoiding the pitfall of “local greenness, global pollution.” A comprehensive LCA should cover the following stages:

1. Raw material acquisition: Assess the environmental impacts of carbon sources, catalysts, and solvents.
2. Synthesis and production: Evaluate energy consumption, water consumption, waste generation, and emissions.
3. Agricultural application: Evaluate environmental release and potential risks during application.
4. Environmental fate and transformation: Assess persistence, degradation, and transformation products in soil and water.
5. Waste management: Evaluate the environmental impacts of final disposal options.

Beyond environmental impacts, techno-economic considerations are also integral to sustainability. These include the production cost of nanomaterials, the feasibility of scaled-up production, product stability, application efficiency, and compatibility with existing agricultural practices. Only when CNTs and CQDs are both environmentally benign and economically viable can they realize their potential in sustainable agriculture. The dual-faced nature of these nanomaterials—therapeutic at low doses yet hazardous at high—is summarized in Figure 4, alongside green synthesis routes that minimize their ecological footprint.

## 7. Potential Synergistic Applications of CNT–CQD Composites in Sustainable Agriculture

### 7.1. Theoretical Basis of Synergy and Composite Construction

CNTs and CQDs each exhibit distinct advantages in sustainable agriculture. Combining them into composite materials holds promise for overcoming the limitations of individual components through synergistic effects. This approach may also introduce new functionalities. This section examines the potential agricultural applications of CNT–CQD composites. It strictly distinguishes between existing agricultural demonstrations and analogies drawn from non-agricultural research. Current knowledge gaps are also identified.

The synergistic effect between CNTs and CQDs originates from the heterojunction formed at their interface. This heterojunction enables efficient charge and energy transfer through photoinduced electron transfer (PET) and Förster resonance energy transfer (FRET) [205]. This synergistic effect is expected to enable more efficient light harvesting, electron transfer, or cargo loading in agricultural applications.

Construction strategies progress from weak to strong interfacial interactions: non-covalent assembly, covalent bonding, and in-situ growth. Each presents trade-offs between binding strength and functional tunability, chosen based on application requirements [206,207,208].

For example, in situ growth enables atomic-level contact and uniform distribution. It therefore offers the possibility of precise control over the interfacial properties of nanomaterials in agricultural applications.

Current strategies form a technical gradient in interface control precision, yet an ideal method combining atomic precision, high CQD loading, and scalability remains elusive.

### 7.2. Potential Applications of Composites in Sustainable Agriculture

CNT–CQD composites hold promise following a “Nutrient Delivery—Environmental Sensing—Plant Growth Regulation” progression. Sensing is currently the most mature area, while delivery and growth regulation remain largely conceptual. Environmental safety assessment underpins all applications [209].

As nanofertilizer carriers, the design exploits functional complementarity: CQD surface groups chelate nutrients for sustained/responsive release, while CNTs facilitate cellular penetration to deliver CQD–nutrient complexes [210]. However, research on such composites is scant; systematic in vivo studies on uptake, translocation, distribution, and metabolism within plants are lacking [211].

Composite Sensing Platforms offer advantages in monitoring soil heavy metals, pesticide residues, and plant hormones. CQDs provide a fluorescence “on-off” response, while CNTs amplify signals and conduct electricity [160,212,213]. For heavy metal detection, CQD fluorescence is efficiently quenched by ions like Hg^2+^, Pb^2+^, Cu^2+^ via static quenching or electron transfer; CNT incorporation further enhances quenching efficiency and sensitivity [214]. Reviews highlight the unique potential of CNT/CQD-based sensors for soil nutrient monitoring and rapid pesticide detection [215].

Environmental Safety remains the paramount bottleneck. Ecotoxicological data focuses overwhelmingly on individual CNTs or CQDs; assessments of CNT–CQD composites are virtually absent. Critical knowledge gaps include composite migration, transformation, degradation in soil; impacts on soil microbial communities; and potential trophic transfer and biomagnification risks. Addressing these is essential before practical deployment.

## 8. Conclusions, Research Gaps, and Future Perspectives

In conclusion, CNTs and CQDs are not substitutes but functionally complementary “nano-agricultural tools.” CNTs anchor the soil-rhizosphere interface, while CQDs target the plant’s internal signaling network. Their synergistic integration, rather than isolated deployment, represents the logical evolution towards “system-integrated” nanoagriculture.

Despite outlining fundamental behaviors, critical gaps hinder rational field design:

(1) Long-Term Fate & Aging Products in Real Environments: Most studies use hydroponics or short-term pot trials. Dynamics of CNT fragmentation, coating degradation, and secondary micro/nano-plastic formation over years in soil are unknown. CQD mineralization rates and metabolite toxicity under field conditions remain understudied.
(2) Molecular Recognition & Interface Specificity: Current evidence relies on correlative transcriptomics/physiology. Direct proof of physical binding between these materials and specific plant receptor proteins (e.g., conformational changes induced by TIR1 binding to CQD carboxyl groups) via structural biology or in-situ force spectroscopy is lacking.
(3) Systemic Behavior Under Multi-Stressor Conditions: Real agroecosystems involve interacting stresses. Current research focuses almost exclusively on single-factor experiments.
(4) Toxicity Mechanisms of CNT–CQD Composites: While Section 7 highlights synergistic potential in sensing/catalysis, the independent behavior of composites at the soil–plant interface—their uptake, translocation, metabolism, and dissipation kinetics—is undocumented. Whether synergy persists amidst high ionic strength and organic matter in real soils is unknown.
(5) Scaling from Model Plants to Staple Crops: Conclusions derive largely from Arabidopsis, lettuce, tomato. Data on full-life cycle effects in staple crops (wheat, rice, maize) are scarce, creating significant uncertainty when extrapolating risk assessments to food security.

Future research should advance along three complementary dimensions: precision, systematization, and sustainability.

(1) Precision through predictive nanomaterial design. Future studies should establish machine learning-driven quantitative structure–activity relationship (QSAR) models that correlate key physicochemical properties of CNTs (e.g., chirality, length, diameter, and surface functionalization) and CQDs (e.g., size, surface charge, heteroatom doping, and functional groups) with crop-specific physiological responses. Integrating high-throughput experimentation with artificial intelligence will enable accurate prediction of nonlinear dose–response relationships and facilitate the development of digital twin platforms capable of recommending optimal nanomaterial formulations for specific crop–soil systems.
(2) Systematic elucidation of plant–nanomaterial interactions. Mechanistic understanding should be strengthened through single-particle, in situ tracking technologies, including synchrotron radiation X-ray fluorescence microscopy (SR-XRF), nano-secondary ion mass spectrometry (NanoSIMS), and single-cell Raman spectroscopy. Coupling these approaches with spatial transcriptomics, proteomics, metabolomics, and ionomics will provide comprehensive insights into nanomaterial transport, cellular localization, molecular responses, and long-term biological effects.
(3) Smart and sustainable agricultural applications. Future efforts should focus on engineering stimuli-responsive nanoplatforms that combine CNTs as structural scaffolds with CQDs as sensing and signaling components. Such systems could detect environmental cues, including drought, salinity, or pathogen attack, and trigger controlled release of nutrients, phytohormones, or protective compounds, enabling proactive rather than passive crop protection. Equally important is the implementation of standardized life cycle-based ecological risk assessment frameworks encompassing raw material sourcing, synthesis, agricultural application, environmental transformation, degradation pathways, and long-term ecotoxicity. Finally, long-term, multi-location field trials under realistic agricultural management practices are essential to validate laboratory findings, evaluate agronomic performance across diverse agroecosystems, and support the safe, scalable deployment of carbon-based nanomaterials in sustainable agriculture. Figure 5 envisions the next-generation ‘system-integrated nanoagriculture’, wherein CNTs and CQDs transcend isolated applications to form functionally complementary nano-toolkits.

## Figures and Tables

**Figure 1:**
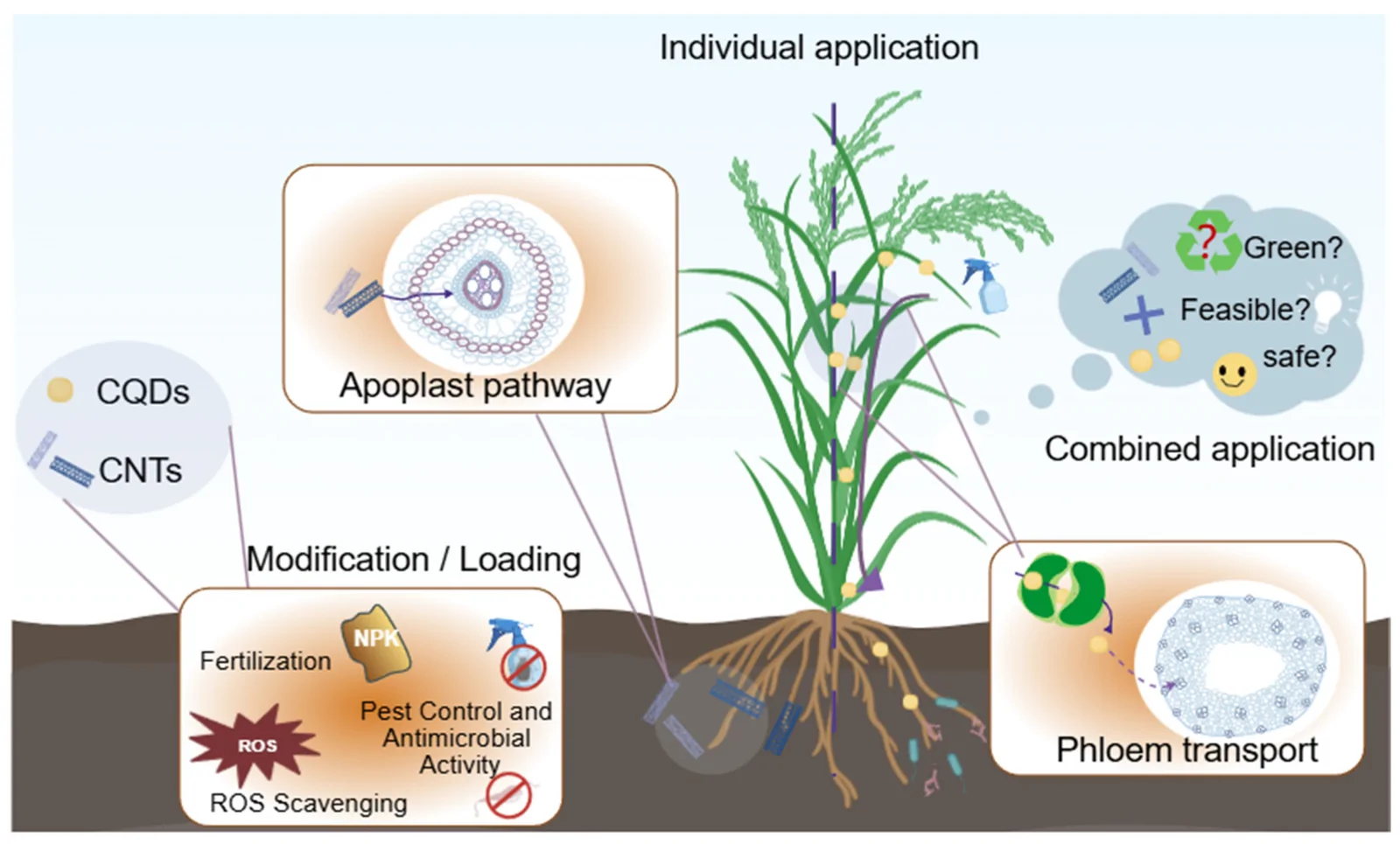
Individual and combined application modes of carbon nanotubes (CNTs) and carbon quantum dots (CQDs) in sustainable agriculture and their core evaluation framework: application strategies and comprehensive assessment indicators, with an emphasis on pre-application modification and multi-functional loading (e.g., fertilization, pest control, antimicrobial activity, and ROS scavenging) on the left. Solid arrows: Experimentally validated pathways; Dashed arrows: Hypothetical mechanisms requiring further verification.

**Figure 2:**
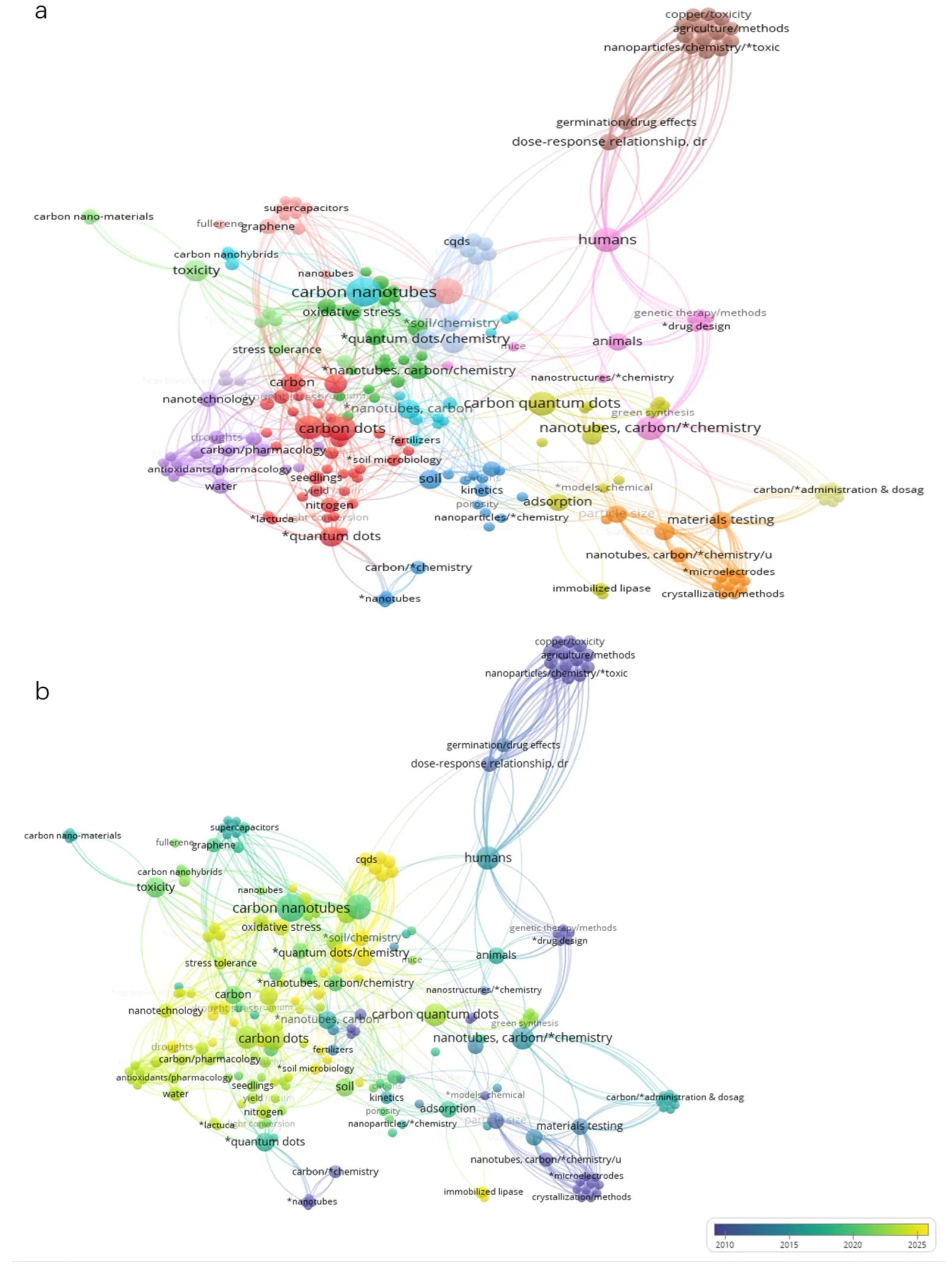
(**a**). Keyword co-occurrence network of the retrieved literature on carbon nanotubes (CNTs) and carbon quantum dots (CQDs) in agriculture (2000–2026). The network was generated using VOSviewer. Node size represents keyword frequency; link thickness indicates co-occurrence strength; colors denote distinct thematic clusters. Note: In the retrieved literature, the terms “carbon dots,” “carbon nanodots,” and “quantum dots” are often used interchangeably with CQDs; in this review, they are uniformly referred to as CQDs. (**b**). Temporal overlay of the keyword co-occurrence network. Node color indicates the average publication year of studies associated with each keyword, from dark blue (2010) to yellow (2025). The map shows a clear temporal shift from toxicity and material characterization toward plant stress mitigation, soil application, and sustainable agriculture. The “*” indicates wildcards used to merge variants of the same keyword stem (e.g., *toxic includes toxic, toxicity, and toxicology; *quantum dots includes quantum dot and quantum dots).

**Figure 3:**
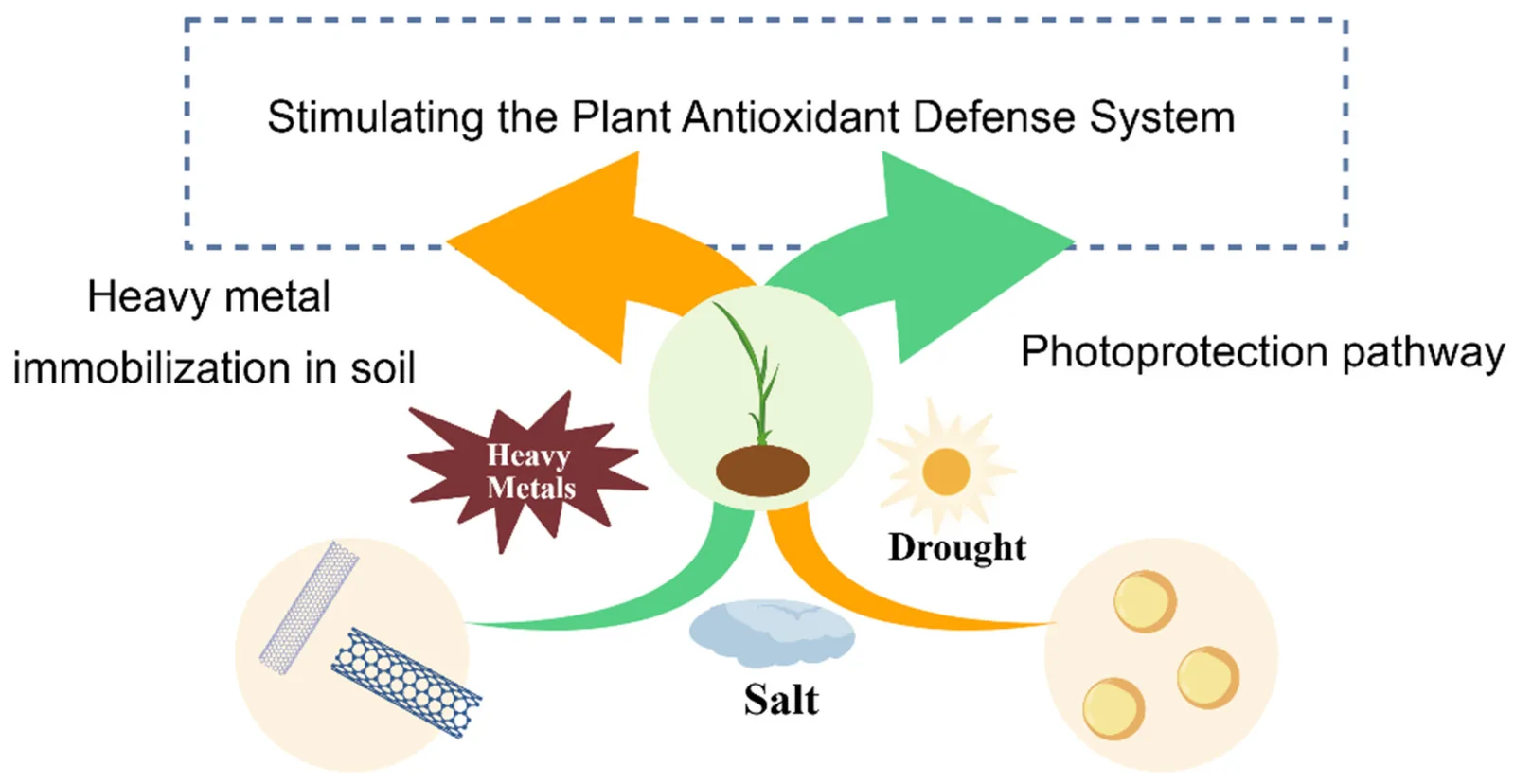
Common mechanisms and divergent strategies of CNTs and CQDs in mitigating heavy metal, drought, and salt stresses.

**Figure 4:**
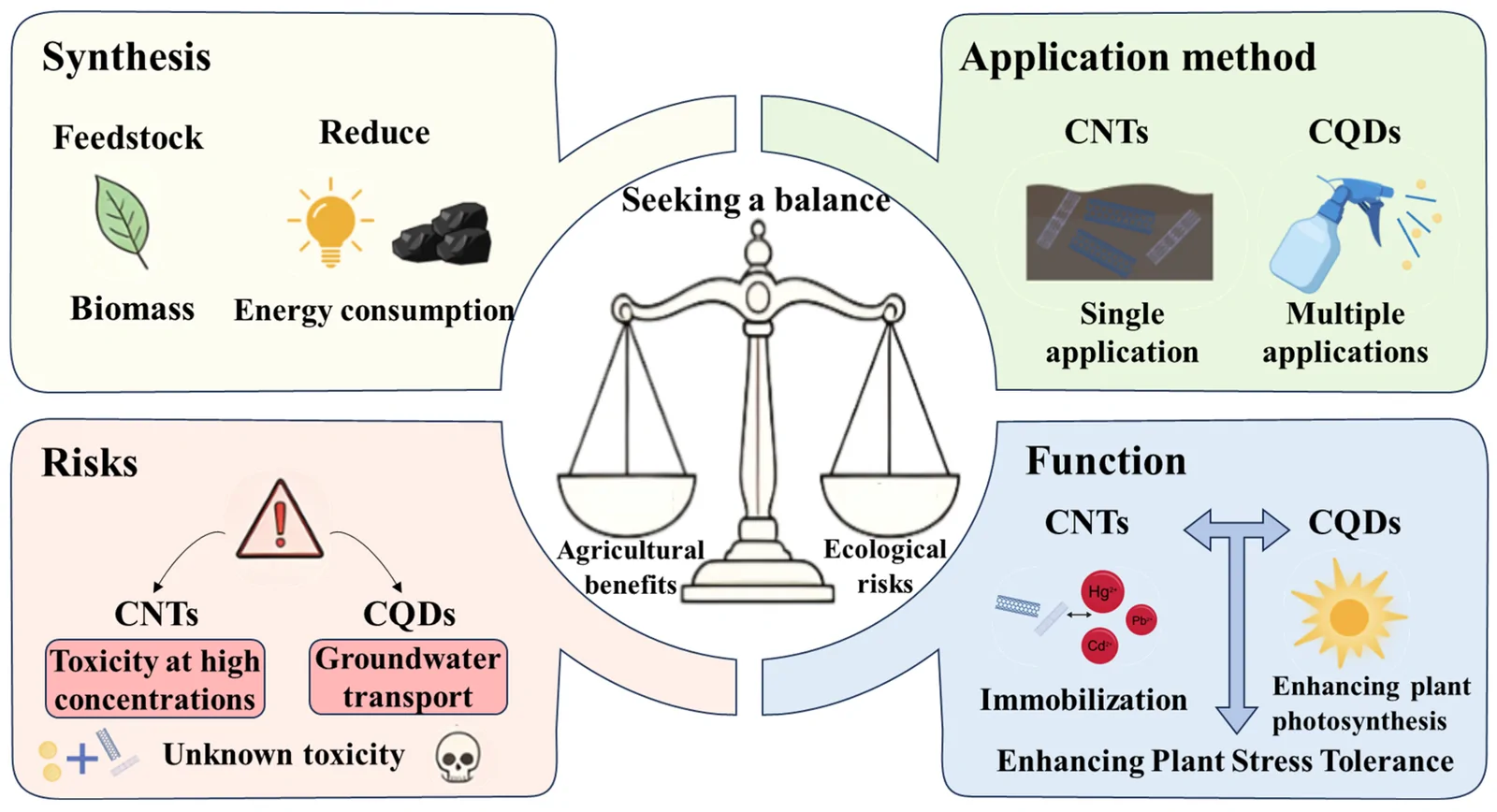
Schematic illustration of the Life Cycle Assessment of biomass-derived carbon nanomaterials (CNTs and CQDs), encompassing feedstock synthesis, application strategies, functional benefits, and potential ecological risks.

**Figure 5:**
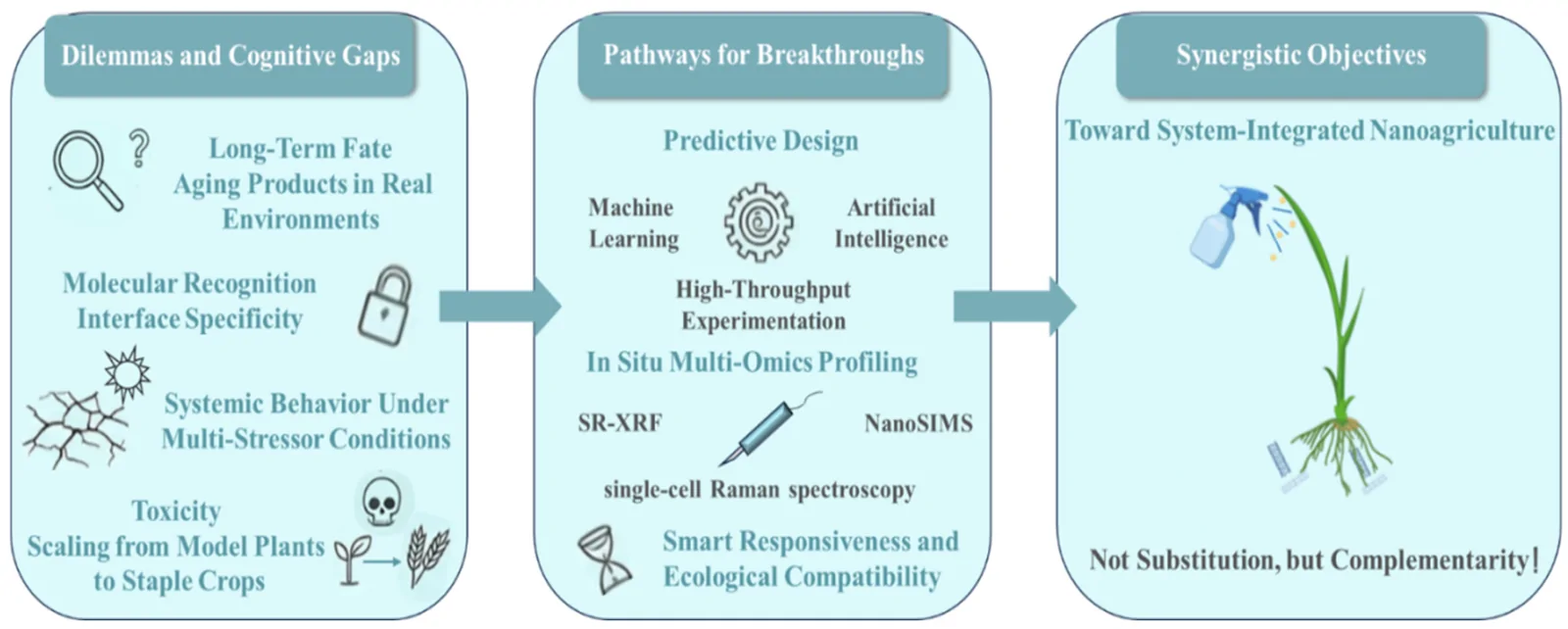
From cognitive gaps to system integration: the logical framework, breakthrough pathways, and synergistic objectives of carbon-based nanomaterials in agriculture.

**Table 1:** Comparison of Structural and Physicochemical Properties of CNTs and CQDs.

Property	Carbon Nanotubes (CNTs)	Carbon Quantum Dots (CQDs)
Dimension & Morphology	1D Tubular	0D Quasi-spherical/Spherical Nanoparticles
Size Range	SWCNTs: Diameter 0.4–3 nm, Length μm-scale; MWCNTs: Outer Diameter ~20–30 nm, Interlayer Spacing 0.34–0.39 nm	Typically <10 nm (mostly 2–8 nm), Tunable
Structural Unit	sp^2^ Carbon Hexagons Rolled, Hollow Core	sp^2^ Carbon Core, Surface-rich O/N Functional Groups (-OH/-COOH/-NH_2_), Often with Surface Molecular Fluorophores
Specific Surface Area	Extremely High	High
Surface Modifiability	Requires pre-oxidation for -COOH/-OH introduction for covalent linkage; strong π–π stacking prone to entanglement	Inherently rich in -COOH/-OH/-NH_2_; Facile aqueous dispersion, functionalization, heteroatom doping (N/P/S/B)
Optical Characteristics	Intrinsically Weak Fluorescence	Strong Fluorescence, Excitation Wavelength Dependent
Electrical/Thermal	Excellent	Weaker than CNTs; Typically Semiconducting/Insulating, Sufficient for Sensing/Optoelectronics
Water Dispersibility	Poor	Good
Synthesis Routes	CVD, Arc Discharge, Laser Ablation	Top-down (Arc Discharge, Laser Ablation, Electrochemical Oxidation); Bottom-up (Hydrothermal/Solvothermal, Microwave, Template Methods)

**Table 2:** Dose–response relationships and potential risks of CNTs and CQDs.

Material Type	Characterization	Crop	Exposure	Dose	Treatment Duration	Endpoints	Adverse Effects	Reference
CQDs	Precursors: citric acid/urea. Morphology: spherical. Particle size: nanoscale. Surface: rich in hydrophilic groups	Soybean	Hydroponics	0–1000 mg·L^−1^	10 d	Photosynthetic efficiency; amino acid biosynthesis	High doses may inhibit growth. A dose-dependent toxicity threshold exists	[192]
	Precursor: guava leaves. Particle size: 6.17 nm	Rice	Seed priming	100–600 ppm	12–36 h	Plant height; root development; grain yield	Inhibitory effects may occur beyond the optimal dose. Long-term ecological accumulation requires assessment	[193]
	Isotope labeling: ^13^C-CQDs. Involved in metabolic transformation	Maize	Foliar spray	0 and 20 mg·L^−1^; 5 mL	14 d	Photosynthesis; sucrose synthesis; TCA cycle intermediates	Potential risk of food-chain transfer. Soil residue effects need further evaluation	[194]
	Polymer functionalization. Surface modification alters biological interactions	Tomato	Seed soaking	0–200 mg·L^−1^	1 h	Biotic stress response indicators; germination rate	Induces unintended biotic stress responses. May interfere with normal immune signaling pathways	[195]
CNTs	Multi-walled carbon nanotubes (MWCNTs)	Barley, soybean, maize	Hydroponic addition	50 µg·mL^−1^	20 weeks	Plant development; biomass; long-term toxicity assessment	No significant toxicity was observed at this concentration. However, physical damage risk may exist at higher concentrations	[196]
	Different solid contents (75%, 95%)	Arabidopsis	Direct addition to culture medium	24.93 µg·mL^−1^, 53.55 µg·mL^−1^	Seedling stage	Gas exchange; photoreaction efficiency; carbon fixation	Inhibits photosynthetic carbon reactions and light reactions. Reduces stomatal conductance	[197]

## Data Availability

No new data were created or analyzed in this study. Data sharing is not applicable to this article.

## Abbreviations

Abbreviation	Full Name
0D	Zero-dimensional
1D	One-dimensional
2D	Two-dimensional
ABA	Abscisic acid
AKT	Potassium channel
APX	Ascorbate peroxidase
ARF	Auxin response factor
ATPS	ATP sulfurylase
AUX/IAA	Auxin/indole-3-acetic acid
B-CQDs	Blue-emissive antioxidant carbon quantum dots
CAT	Catalase
CBM	Conduction band minimum
CLSM	Confocal laser scanning microscopy
CNMs	Carbon-based nanomaterials
CNTs	Carbon nanotubes
CQDs	Carbon quantum dots (unified term; includes undoped, doped, nutrient-loaded, and hybrid forms)
*CRL1*	Crown rootless 1 (rice gene)
CS	Cysteine synthase
CVD	Chemical vapor deposition
ΔG^0^	Gibbs free energy change
ETR	Electron transport rate
ETC	Electron transport chain
f-MWCNTs	Functionalized multi-walled carbon nanotubes
FA	Fulvic acid
FRET	Förster resonance energy transfer
Fv/Fm	Maximum quantum efficiency of photosystem II
GO	Graphene oxide
GPX	Glutathione peroxidase
GR	Glutathione reductase
GS	Glutamine synthetase
HA	Humic acid
HDA	Electronic coupling matrix element
kET	Electron transfer rate constant
LCA	Life cycle assessment
LOD	Limit of detection
LUE	Light use efficiency
MDA	Malondialdehyde
MWCNTs	Multi-walled carbon nanotubes
MWCNT-COOH	Carboxyl-functionalized multi-walled carbon nanotubes
MWCNT-NH_2_	Amine-functionalized multi-walled carbon nanotubes
N-CQDs	Nitrogen-doped carbon quantum dots
N,P-CQDs	Nitrogen and phosphorus co-doped carbon quantum dots
NanoSIMS	Nano-secondary ion mass spectrometry
NIR	Near-infrared
NRT	Nitrate transporter
PAR	Photosynthetically active radiation
PET	Photoinduced electron transfer
PEDOT	Poly(3,4-ethylenedioxythiophene)
PGPR	Plant growth-promoting rhizobacterium
PL	Photoluminescence
PNDs	Polyacrylic acid-modified nitrogen-doped carbon dots
POD	Peroxidase
PP2C	Protein phosphatase 2C
PPy	Polypyrrole
PSI	Photosystem I
PSII	Photosystem II
p-MWCNT	Pristine multi-walled carbon nanotube
PYR/PYL	Pyrabactin resistance/pyrabactin resistance-like
QSAR	Quantitative structure–activity relationship
RCDs	Red-emissive carbon dots (red-emissive CQDs)
ROS	Reactive oxygen species
RSA	Root system architecture
RT-PCR	Reverse transcription polymerase chain reaction
Rubisco	Ribulose-1,5-bisphosphate carboxylase/oxygenase
RWC	Relative water content
SiR	Sulfite reductase
SnRK2	Sucrose nonfermenting 1-related protein kinase 2
SOD	Superoxide dismutase
SR-XRF	Synchrotron radiation X-ray fluorescence microscopy
SWCNTs	Single-walled carbon nanotubes
TEM	Transmission electron microscopy
TIR1/AFB	Transport inhibitor response 1/auxin signaling F-box
UCPL	Upconversion luminescence
UV-Vis	Ultraviolet-visible
VBM	Valence band maximum
WUE	Water use efficiency
ΦII	Quantum efficiency of photosystem II
λ	Reorganization energy
